# A novel *TPM2* gene splice-site mutation causes severe congenital myopathy with arthrogryposis and dysmorphic features

**DOI:** 10.1007/s13353-016-0368-z

**Published:** 2016-10-10

**Authors:** Magdalena Mroczek, Dagmara Kabzińska, Krystyna H. Chrzanowska, Maciej Pronicki, Andrzej Kochański

**Affiliations:** 1 Neuromuscular Unit, Mossakowski Medical Research Centre, Polish Academy of Sciences, A. Pawi ńskiego 5, 02-106 Warsaw, Poland; 2 Department of Medical Genetics, The Children’s Memorial Health Institute, Al. Dzieci Polskich 20, 04-730 Warsaw, Poland; 3 Department of Pathology, The Children’s Memorial Health Institute, Warsaw, Poland

**Keywords:** *TPM2* gene, Unspecified congenital myopathies, Whole exome sequencing

## Abstract

To date, only two splice-site mutations within the *TPM2* gene have been shown to be causative for congenital myopathies. While the majority of *TPM2* gene mutations are causative for nemaline myopathy, cap disease or distal arthrogryposis, some mutations in this gene have been found to be associated with non-specific congenital myopathy. We report on a patient with such an unspecified congenital myopathy associated with distinctive facial dysmorphic features and distal arthrogryposis. Using the whole exome sequencing (WES) approach we were able to identify a novel heterozygous splice-site mutation within the *TPM2* gene, showing the utility of WES in molecular diagnostics of congenital myopathies without recognizable morphological hallmarks.

## Introduction

The tropomyosins (TPMs), encoded by four genes (α, β, γ, and δ), are involved in a wide spectrum of cellular processes (Gunning et al. 2005). In the striated and smooth muscle, TPMs are key regulators of calcium–dependent muscle contraction. TPM binds to actin filaments, which become permissive for myosin II interactions (Gunning et al. 2005). TPM2 is expressed mainly in slow type-1 muscle fibers (Tajsharghi et al. 2012).

Mutations in the β-tropomyosin gene (*TPM2*) have been shown to be associated with a spectrum of phenotypes ranging from a pure distal arthrogryposis (DA) to cap disease and nemaline myopathy (Tajsharghi et al. 2012).

Though almost all *TPM2* gene mutations are inherited in an autosomal dominant manner, one homozygous nonsense *TPM2* mutation was found in a patient with a very severe nemaline myopathy (Monnier et al. 2009). Two *TPM2* mutations, *i.e.*, c.349G>A, p.Glu117Lys and, c.364G>A; p.Glu122Lys, were identified in an unspecified congenital myopathy, diagnosed in the patients without specific structural alterations in the muscle biopsy specimen (Brandis et al. 2008; Donner et al. 2002). Some of the TPM2 gene mutations were reported to be associated with congenital fiber type disproportion (CFTD). Among 27 *TPM2* mutations reported to date in congenital myopathies, only two, *i.e.*, c.240+2T>C and c.240+5G>A, are splice-site mutations (Marttila et al. 2014). Interestingly, a recurrent *TPM2* mutation, c.20_22delAGA, p.Lys7del, is responsible for two distinct clinical entities, *i.e.*, core-rod myopathy and distal arthrogryposis type 7 (Davidson et al. 2013). In this study we report on a patient manifesting a combined phenotype of facial dysmorphism, unspecified congenital myopathy, and distal arthrogryposis.

## Patient and methods

### Muscle biopsy analysis

Frozen sections of a *quadriceps femoris* muscle sample were stained with hematoxylin and eosin, modified Gomori trichrome, oil red O, and picrosirius red. Histochemical reactions included: succinate dehydrogenase; NADH dehydrogenase; cytochrome c oxidase, myosin ATP-ase at pH 4.3; 4.6; and 9.4. An immunohistochemical controlled panel for dystrophin, beta sarcoglycan, spectrin, and merosin was also performed.

### Molecular analysis

The exome sequencing in the proband was performed in line with the protocol from *Illumina*’s *TruSeq* Exome Enrichment Guide. Exome capture was performed on the genomic DNA with a *SureSelect* Human All Exon 50 Mb Kit (*Agilent Technologies*) and the*HiSeq 2000* instrument (*Illumina*). Exome sequencing was performed by the firm *Intelliseq sp. z o.o.*, based in Cracow. The sequence reads were analyzed using an *Illumina* pipeline, with reads processed by *Picard* and aligned to a human reference sequence (GRCh37) using *Bowtie 2* (Langmead and Salzberg 2012). *SAMtools* were used to obtain BAM files for analyzing samples (Li and Durbin 2009) and removing duplicated reads. Variant calling was then performed using a Genome Analysis Toolkit (GATK) (McKenna et al. 2010). NGS variants analysis were done on the Galaxy platform (Blankenberg et al. 2010; Giardine et al. 2005; Goecks et al. 2010). The data were filtered by genotype: the proband as heterozygous or homozygous alternative and parents as homozygous reference or heterozygous, with 12 other control probes being used as homozygous reference. Data were then annotated with dbSNP and SnpEff, and common sequence variants were filtered out. The *SIFT* tool (Kumar et al. 2009; Ng and Henikoff 2001, 2002, 2003) and *SnpSift* (Cingolani et al. 2012) were used for conservation analysis and effect prediction in relation to SNPs. Finally, filtering for rare and probably protein deleterious variants was carried out. The presence of WES detected variants was confirmed by Sanger sequencing. To estimate the influence of the mutation on the RNA transcript, total RNA was isolated from leukocytes and after reverse transcription the fragment of *TPM2* cDNA covering exons 2, 3, and 4 was amplified using designed primers (on request), and sequenced directly. The length of the cDNA fragment was measured on GeneScan.

The CGH analysis was performed using the *Agilent* Human Genome G3 Sure Print 8 × 60 Microarray (*Agilent Technologies*, USA) with resolution over 100 kb.

DNA samples obtained from 100 healthy controls (200 chromosomes) were screened for mutation c.374+2T>C in the *TPM2* gene, using the RFLP-PCR approach with *Aci* I restriction enzyme.

## Results

### Clinical features of the proband

The female proband was born to young, unrelated parents (a mother and father aged 29 and 32 years, respectively), after two miscarried pregnancies.

In the mother, the homozygous c.1298A>C, p.Glu433Ala mutation in the *MTHFR* (1p36.3) gene was identified and antiphospholipid syndrome diagnosed in the regional hospital.

A non-invasive 1st-trimester prenatal screening was carried out since nuchal translucency and polyhydramnios were revealed by ultrasound scanning. The PAPP-A test indicated an increased risk of Down syndrome. Prenatal cytogenetic analysis revealed a normal female karyotype (46,XX). Smith–Lemli–Opitz syndrome was excluded by analysis of levels of amniotic fluid 7 and 8-dehydrocholesterol (7/8-DHC). Magnetic resonance imaging (MRI) performed at 24 weeks of gestation suggested a coccygeal bone defect, cloacal anomaly, and bilateral clubfoot.

Cesarean section was performed at 35 weeks of gestation, due to fetal condition complicated by intrauterine growth retardation and polyhydramnios. Her birth weight was 1900 g (-1.1 SD; 13th centile) and the Apgar score was 4 at 1st minute. The baby was admitted to the neonatal intensive care unit (NICU) in the regional hospital for mechanical ventilation, because the clinical course was seriously complicated by pulmonary hypertension and generalized hypotonia. The baby did not demonstrate either a suck reflex or a swallowing reflex. On admission, dysmorphic facial features were recorded such as hypertelorism and prominent eyes, as well as hypertrichosis of the sacroiliac region, contractures of wrists, knees, fingersand toes, and bilateral clubfoot. Transcranial ultrasonography showed intraventricular hemorrhage (IVH) of I and II degree, and asymmetry of the ventricles. MRI indicated corpus callosum hypoplasia and shallow orbits. An X-ray of the entire body (babygram) did not show symptoms of generalized genetic bone disease, while an X-ray of the thorax revealed a raised diaphragm. The methylation test for Prader-Willi syndrome (PWS) showed normal pattern of methylation in chromosome 15q11-13 region. Deletions of exons 7 and 8 of the *SMN1* gene were excluded.

On the 93rd day of life she was admitted to the Intensive Care Unit at the Children’s Memorial Health Institute (CMHI) in Warsaw, where a tracheotomy was performed due to a persistent generalized hypotonia and necessity of permanent mechanic ventilation. A muscle biopsy performed at the age of 7 months revealed congenital fiber type disproportion (CFTD). Auditory evoked potentials (AEP) showed bilateral sensorineural hypoacusis. The patient was nourished using an NG-tube. She was discharged home after a four-month hospitalization in a stable state, and home mechanical ventilation (HMV) was recommended.

At 10 months of life the baby manifesting poor weight gain (5450 g; −4.5 SDS) was readmitted to the CMHI and a gastrostomy tube (G-tube) was inserted. At the age of 11 months her growth was still severely retarded: length 73 cm (−2.67 SDS), head circumference 44.5 cm (−2.24 SDS), weight 6.4 kg (−3.16 SDS), and BMI 12.1 (−3.13 SDS), although she put on weight substantially.

**Fig. 1:**
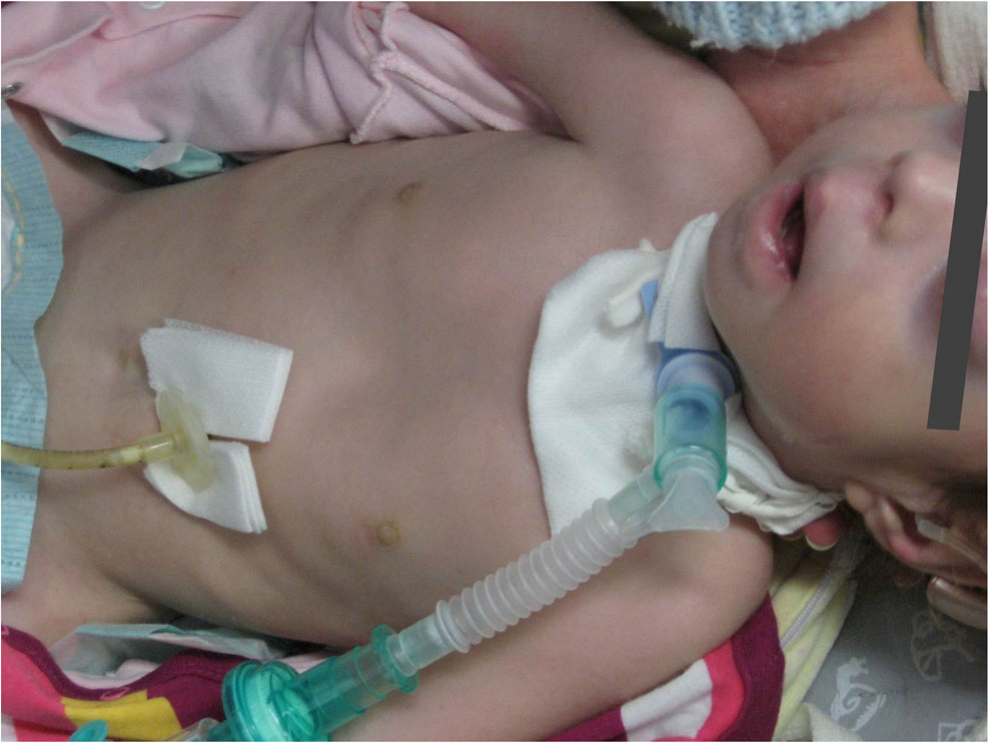
The proband aged 13 months, manifesting dysmorphic craniofacial features: elongated face with bitemporal narrow forehead, low frontal hairline, widely spaced and prominent eyes, short nose with depressed nasal bridge, underdeveloped nasolabial fold, and open mouth

At the age of 13 months, despite intensive physiotherapy, contractures in the small joints of the hands and feet (with overlapping fingers and toes), as well as in the knee and at the hip joints were present. There was reduced flexion of the wrist and elbow joint. Both patellar reflexes were present. The child presented with pectus excavatum and generalized hypotonia. The patient had a hearing aid due to the sensorineural hypoacusis in both ears. Mechanical ventilation was needed almost constantly (Fig. 1). The patient died suddenly aged 2 years and 8 months.

### Quadriceps femoris muscle biopsy

**Fig. 2:**
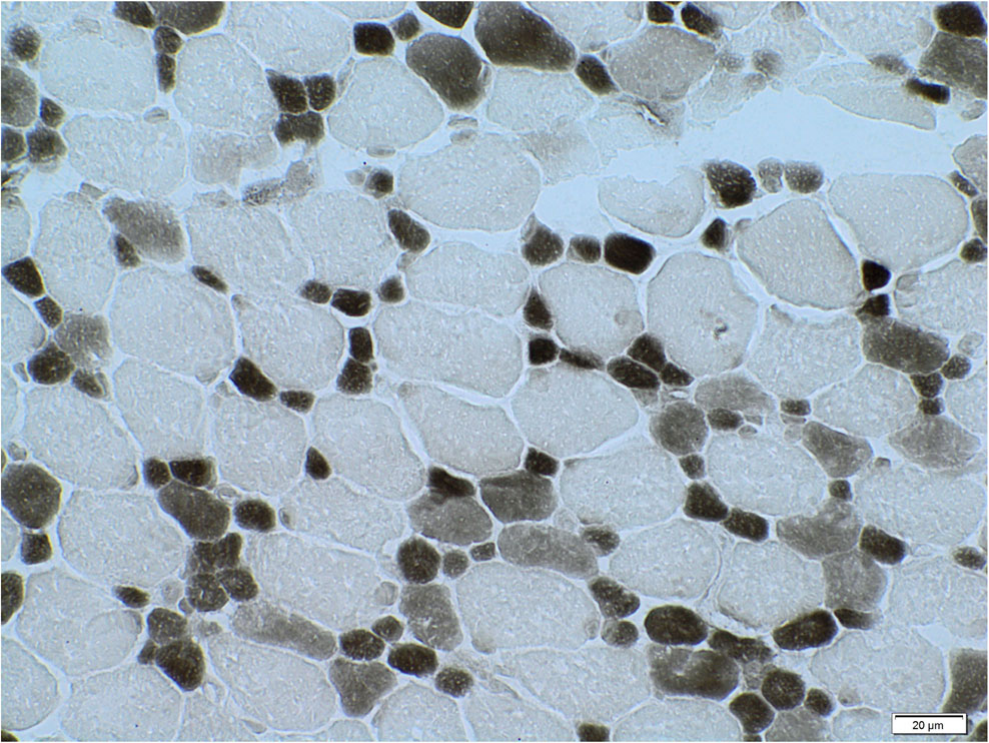
Skeletal muscle biopsy, myosin ATP-ase at pH 4.3. Original magnification 600×. Congenital fiber type disproportion pattern

Skeletal muscle revealed considerable bimodal variability of muscle-fiber diameters (4–25 μm) with selective smallness of type-1 fibers. No other essential abnormalities were found. The result was concluded to be a congenital muscle fiber type disproportion (Fig. 2).

### Molecular analysis

No pathogenic mutations in *LMNA*, *IGHMBP2*, *EGR2*, *MTMR2*, and *SBF2* genes were found using the Sanger sequencing method.

A whole-exome sequencing analysis revealed a novel splice-site c.374+2T>C mutation within the *TPM2* gene in the proband (confirmed by a Sanger method). The mutation was not present in 100 healthy controls (200 chromosomes) from the Polish population.

**Fig. 3:**
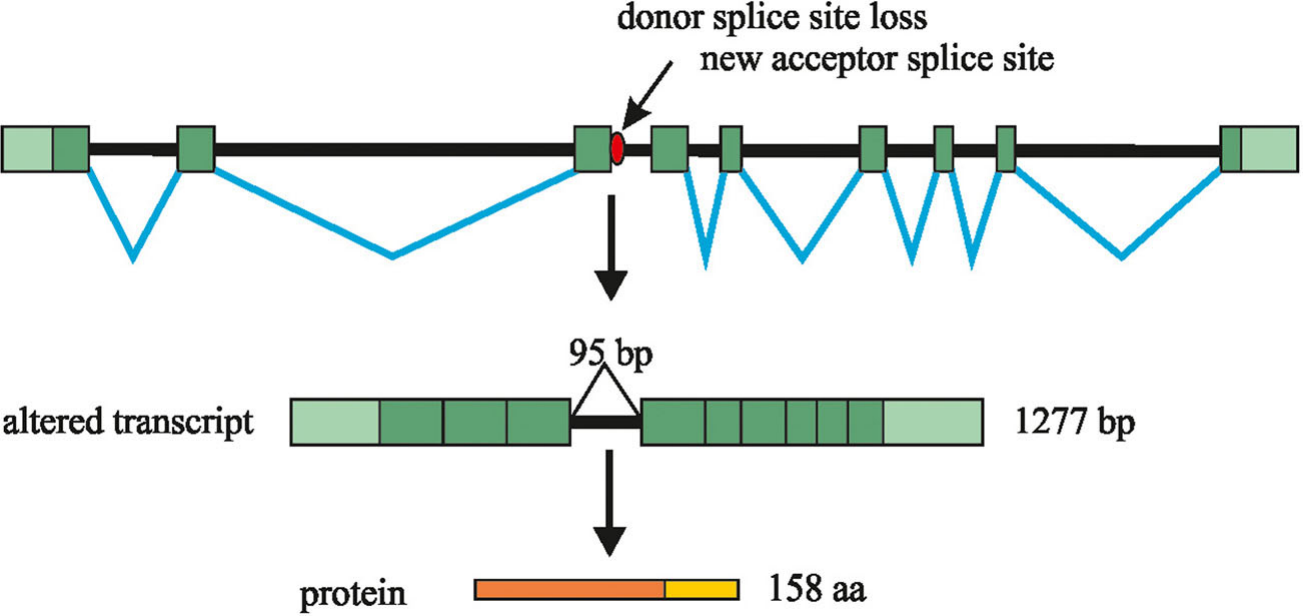
Schematic diagram showing the effect of the c.374+2T>C mutation on mRNA splicing of the *TPM2* gene. The c.374+2T>C mutation results in the abolition of the donor splice-site. The 3^rd^ intron of the *TPM2* gene is present in the aberrantly spliced transcript. Finally the frameshift mutation results in a premature stop codon

Gene Scan analysis of the *TPM2* cDNA fragment in the proband revealed two peaks, which correspond with two products, *i.e.*, a longer RNA transcript of about 463 bp and a normal–length, wild-type RNA transcript of about 368 bp. In the proband’s parents only one peak (368 bp) corresponding with the normal wild-type RNA transcript was detected. The sequencing of the cDNA in the proband showed that the RNA transcript in addition contains the whole sequence of the 3^rd^ intron of the *TPM2* gene (of 95 bp) (Fig. 3).

Bioinformatics analysis indicated that a longer RNA transcript results in frameshift at a codon 125, and a premature stop codon 34 aa downwards. Thus, by in silico translation, the mutation results in a truncated TPM2 protein (p.Gly125TrpfsX34).

A whole-exome sequencing analysis (with sequence variants filtered as described in Methods) carried out in the proband revealed rare sequence variants in more than 200 genes, including these involved in the pathogenesis of neuromuscular disorders.

## Discussion

In this study we document a patient with a severe form of unspecified congenital myopathy, with arthrogryposis and facial dysmorphic features, in whom a new splice-site mutation within the *TPM2* gene was found. Functional studies of the *TPM2* c.374+2T>C mutation confirmed the pathogenic effect of this mutation.

The molecular diagnostics process in the proband proved extremely difficult, due to a lack of any specific ultrastructural features for the congenital myopathy. Two mutations within the *TPM2* gene have been found previously in patients manifesting CFTD upon muscle biopsy. The authors suggested that, especially in the case of muscle biopsies taken at very young ages (5, 6 or 8 months), no specific abnormalities may be found (Marttila et al. 2014). Moreover, Lehtokari reported on a patient presenting cap structures at age 33 years, in the second muscle biopsy (Lehtokari et al. 2007). Given that accumulation of mutated proteins occurs over time, it is possible that, in very young children in particular, no specific alterations in a muscle specimen may yet be present. Our observations do fit accurately with this hypothesis. Interestingly, therefore, with a classical approach to genetic analysis directed by the ultrastructure in the congenital myopathy, the molecular diagnostics process could be misdirected. In such cases, whole-exome sequencing represents a very useful approach not influenced by the histopathological findings.

Only two splice-site mutations within the *TPM2* gene have been reported to date in patients with congenital myopathy. The pathogenic character of the c.374+2T>C mutation in the *TPM2* gene has been documented by us. First, the c.374+2T>C mutation is associated with the phenotype of CFTD as previously reported in patients with mutations in the *TPM2* gene (Marttila et al. 2014). Second, the c.374+2T>C mutation is located in a highly conservative position which is critical to an appropriate splicing process.

The c.374+2T>C mutation was not detected in the control group. Moreover, the c.374+2T>C mutation was selected in the algorithms of the whole-exome sequencing as the strongest *de novo* sequence variant among thousands of variants detected. Using the WES approach we have also checked other genes associated with neuromuscular disorders, in which no mutations have been detected. Since the procedure used by us was not a single-gene analysis, we have evidence for the pathogenic nature of the c.374+2T>C mutation. Moreover, in the analysis of the cDNA, we have shown that the c.374+2T>C mutation results in an aberrantly spliced transcript containing the 3^rd^ intron of the *TPM2* gene. Typically for other *TPM2* mutations, the c.374+2T>C sequence variant occurs in a *de novo* configuration and is inherited as an autosomal dominant fashion.

A combined phenotype of the congenital myopathy, distal arthrogryposis and facial dysmorphic features, we observed in the patient may be associated with a pleiotropic effect of the c.374+2T>C mutation.

On the other hand, we cannot exclude that the additional point mutations found using our WES approach have any modifying effect.

To conclude, our study documents the phenotype associated with a new heterozygous c.374+2T>C mutation within the *TPM2* gene segregating with an unspecified congenital myopathy accompanied by facial dysmorphism and arthrogryposis. This study points to the utility of the WES approach, especially in patients not manifesting with any specific morphological features, *i.e.*, patients in which the muscle biopsy is taken very early. The pathogenic effect of the c.374+2T>C mutation has been established in relation to many lines of evidence which were available. We have shown that WES approach should be especially recommended in the patients manifesting with an overt phenotype of congenital myopathy without any specific features in the electron microscope analysis.
